# Do consanguineous parents of a child affected by an autosomal recessive disease have more DNA identical-by-descent than similarly-related parents with healthy offspring? *Design of a case-control study*

**DOI:** 10.1186/1471-2350-11-113

**Published:** 2010-07-16

**Authors:** Marieke E Teeuw, Lidewij Henneman, Zoltan Bochdanovits, Peter Heutink, Dirk J Kuik, Martina C Cornel, Leo P ten Kate

**Affiliations:** 1 Department of Clinical Genetics, Section Community Genetics, EMGO Institute for Health and Care Research, VU University Medical Center, Amsterdam, The Netherlands; 2 Department of Clinical Genetics, Section Medical Genomics, VU University Medical Center, Amsterdam, The Netherlands; 3 Department of Epidemiology and Biostatistics, VU University Medical Center, Amsterdam, The Netherlands

## Abstract

**Background:**

The offspring of consanguineous relations have an increased risk of congenital/genetic disorders and early mortality. Consanguineous couples and their offspring account for approximately 10% of the global population. The increased risk for congenital/genetic disorders is most marked for autosomal recessive disorders and depends on the degree of relatedness of the parents. For children of first cousins the increased risk is 2-4%. For individual couples, however, the extra risk can vary from zero to 25% or higher, with only a minority of these couples having an increased risk of at least 25%. It is currently not possible to differentiate between high-and low-risk couples. The quantity of DNA identical-by-descent between couples with the same degree of relatedness shows a remarkable variation. Here we hypothesize that consanguineous partners with children affected by an autosomal recessive disease have more DNA identical-by-descent than similarly-related partners who have only healthy children. The aim of the study is thus to establish whether the amount of DNA identical-by-descent in consanguineous parents of children with an autosomal recessive disease is indeed different from its proportion in consanguineous parents who have healthy children only.

**Methods/Design:**

This project is designed as a case-control study. Cases are defined as consanguineous couples with one or more children with an autosomal recessive disorder and controls as consanguineous couples with at least three healthy children and no affected child. We aim to include 100 case couples and 100 control couples. Control couples are matched by restricting the search to the same family, clan or ethnic origin as the case couple. Genome-wide SNP arrays will be used to test our hypothesis.

**Discussion:**

This study contains a new approach to risk assessment in consanguineous couples. There is no previous study on the amount of DNA identical-by-descent in consanguineous parents of affected children compared to the consanguineous parents of healthy children. If our hypothesis proves to be correct, further studies are needed to obtain different risk figure estimates for the different proportions of DNA identical-by-descent. With more precise information about their risk status, empowerment of couples can be improved when making reproductive decisions.

## Background

The offspring of consanguineous relations have an average increased risk of 2-4% of congenital/genetic disorders and early mortality. However, on an individual level the exact risk figure can vary to a great extent.

### Global prevalence

The children of consanguineous couples represent a considerable group, since an estimated 10.5% of all children worldwide have consanguineous parents [1]. This frequency is, however, very unevenly distributed between countries. In some countries the current percentage of consanguineous marriages is higher, and may even exceed 50%, while in many others the percentage does not surpass 1% [2,3]. Worldwide, every year over 130 million infants are born [4], which leads to the conclusion that the considerable number of 13.5 million of those children have consanguineous parents.

### Autosomal recessive (AR) inheritance

A child affected by an AR disease has inherited a pathological allele from both parents who are carriers of such an allele. If both parents are carriers, all of their children have a 25% chance of being affected.

### Risk is proportional to degree of relatedness of the parents

The risk of being a carrier couple is on the one hand proportional to the frequency of a pathological allele in the population, and on the other hand proportional to the coefficient of inbreeding (F), which is defined as the probability that a child inherits two identical copies of an allele from one or more common ancestors. The closer the partners of a consanguineous couple are related, the greater the chance that they will have genetic information identical-by-descent (IBD). When the amount of DNA-sharing increases, this also increases the chance of sharing a particular pathological allele IBD and therefore the chance of having affected offspring with an AR disease. Theoretically, the likelihood that an allele passed on to the next generation will be an identical copy of the allele of a common ancestor passed on by the other partner is 1/16 (F = 1/16) for first cousins' offspring, whereas for second cousins' offspring, this is 1/64. For unions less closely related than second cousins, the risk of having an affected child is only marginally increased.

### Burden

Studies among first-cousin couples, the most prevalent type of consanguineous marriage, show that the excess risk for their offspring of having a significant birth defect ranges from 1.7-2.8% [5]. The risk for mortality in early life (i.e. from six months of gestation to an average of ten years of age) in the offspring of first-cousin marriages is estimated at 3.5% [1]. For this latter figure it remains difficult to control for the effects of non-genetic variables. Causes of mortality that are related to other (sociodemographic) variables, like maternal illiteracy, maternal age and birth interval, may, in themselves, lead to a higher rate of neonatal and early childhood mortality and could be confounders [1,6]. Considering that over 10% of all children worldwide have consanguineous parents, combined with the excess risk of 2-4% per first-cousin couple, the conclusion can be drawn that the global burden of pre-reproductive mortality and morbidity for the children and their families is substantial. The proportion of first-cousin marriages among consanguineous couples is estimated to be at least 70% [A.H. Bittles, personal communication]. From this number we can infer that the extra number of affected children born to first-cousin parents is approximately 190,000 to 380,000 each year. The total number of cases due to consanguinity, however, must be higher, since our estimate does not include the affected children born to consanguineous parents who are related in another way.

### Only the minority of consanguineous couples have an increased risk

If one compares the 2-4% additional risk of congenital/genetic disorders and/or early death in children of a first-cousin couple, to the 25% risk of a couple in which both man and wife are carriers of an AR disorder, one has to conclude that a maximum of 8-16% of all first-cousin couples are at high risk (25%; or higher in case of carriership of more than one disorder), while at least 84-92% of all first-cousin couples have a normal risk, comparable to unrelated parents.

### Risk assessment in practice

When a consanguineous couple is referred for risk assessment, e.g. to a clinical genetics centre, best practice prescribes that a thorough family history will be taken [5]. For an average non-consanguineous couple, a risk of 2-3% of having a child with a genetic/congenital disorder is present. For a first-cousin couple, an additional risk of 2-4% should be added to this basic risk. The risk can further increase if a family history for a genetic disorder exists. A more precise risk estimate for that particular disease can then be assessed by risk calculation or - if possible and desired - by carrier testing. If the population of origin of the couple is known to have a high risk of a specific recessive disorder, carrier screening could be offered as well.

A typical characteristic for AR disorders is the fact that frequently there are no previous affected members within the family, since affected family members most often can be found in only one sibship. When there is no history of diseases in the family of a consanguineous couple that comes for preconception counselling, there is still an additional average risk close to 2-4%. However, it is currently not possible to determine who is at 25% (or more) risk, and who has no increased risk. Given this uncertainty, being able to give a more precise risk figure could have important consequences for counselling.

### Stochastic variation

The actual amount of DNA IBD in children of first cousins can be different from the theoretical 1/16 due to a stochastic variation which is caused by the random recombinations in common ancestral loops. This causes a significant variability between couples with the same F-value [7]. A simulation study performed by Leutenegger et al. also showed that considerable variability in estimates of the coefficient of inbreeding derived from whole genome analyses can be found. For example, at first-cousin level, individuals with an expected F = 0.0625 can have from 0.03-0.12 of their genome IBD [8]. Clearly, this variability could significantly alter the probability that a recessive disease gene will be expressed.

### Variation by hidden ancestral loops

A difference in DNA-sharing may also be present while comparing couples with a similar inbreeding coefficient when the estimated coefficient is based on limited available genealogical data. Distant consanguineous loops often remain unknown, which can lead to an underestimation of the inbreeding coefficient [9]. Genealogy-based studies have indicated that after 3-4 generations of cumulative inbreeding and with multiple loops of consanguinity, as would occur in many highly inbred communities, the progeny of first-cousin unions may have F values up to 0.1484, which likewise would be expected to significantly influence recessive gene expression [10]. The variability in DNA sharing in practice was also shown by Woods et al. who studied children with AR disorders whose parents were consanguineous [11]. By using SNP analysis, they found that in individuals with a recessive disease whose parents were first cousins, on average 11% of their genomes were homozygous, as opposed to the 6.25% one would expect.

### Hypothesis

On the basis of the above-mentioned considerations and observations, we hypothesize that consanguineous parents of a child with an AR disorder will have more DNA IBD than similarly-related parents who have only had healthy children. This hypothesis leads to the objective of our present study, namely to establish whether the amount of DNA IBD in partners of consanguineous couples with a child affected by an AR disease is indeed increased compared to its proportion in partners of consanguineous couples who have healthy children only. If so, this result might be applied to improve risk assessment in consanguineous couples.

## Methods/Design

This project is designed as a case-control study in which we test whether consanguineous partners (cases) with children affected by AR diseases indeed share more DNA, IBD, than consanguineous partners (controls) who are believed to be related to the same degree, but only have healthy children. We will do this by making use of genome wide SNP analysis. For this study, approval was obtained from the Medical Ethics Committee of the VU University Medical Center.

### Matching

The cases and controls will be matched as closely as possible to assure that allele frequencies will be valid for both cases and controls, and to decrease the possibility of false positive or false negative results caused by hidden consanguinity in previous generations. The best way to do this is by restricting the search for controls to the same family, but if no suitable controls can be identified, we will search for controls from the same clan or ethnic origin.

### Criteria for inclusion and exclusion

A case couple will only be included when the AR nature of the disorder in the offspring is beyond doubt and the disorder has not occurred in the family before. The exact nature of the AR disorders in the children of the cases is irrelevant, as we will only be testing for the proportion of DNA-sharing irrespective of which regions of the genome are identical. For this reason it is possible to combine results from studies conducted in countries and populations with different AR disease spectra. The inclusion of control couples is restricted to couples whose offspring are not only free from known AR disorders but also from other diseases in which a role for homozygosity cannot be excluded. Control couples should have at least three healthy children. When several control couples are available for a given case, maximum contrast between cases and controls can be achieved by selecting the controls with the highest number of children.

### Numbers needed

Due to lack of knowledge of the exact means and variances in identical DNA in the two groups, our power calculation is based on the assumption that for first-cousin couples, who theoretically have 0.125 of their genome IBD, a standard deviation of 0.0625 will apply. If half a standard deviation or more is considered a relevant effect size, we expect to have sufficient power (90%) by sampling a group of 100 cases and 100 controls. A possible loss of 15% is taken into consideration in this calculation.

### Ascertainment

The recruitment of cases will be done in different medical centres in our country (the Netherlands) and elsewhere. We will locate the consanguineous parents of children with an AR disorder through their treating physicians. After receiving extensive information from the researcher, informed consent is obtained. Control couples, if present in the same families, will be asked to participate by an invitational letter given to them by their participating family members (the cases), or by recruiting them from the same clan or tribe.

A case and control pair will preferably have the same inbreeding coefficient, but this is not essential, since this can be corrected for in the calculations.

### Pedigree information and family history

Information will be obtained on the identity of all first-degree-to third-degree family members of both partners of the index pair. This implies that we try to identify the great-grandparents of the index pair and all first cousins. Family members include the deceased, still-born and miscarriages. For every individual in the family tree, the health status will be carefully noted. A family tree will be drawn and saliva will be obtained from the pair. To increase the amount of information that can be generated from the DNA of the cases and controls, we will also sample DNA from the children if possible, or from other family members, e.g. grandparents of the children. An inbreeding coefficient will be calculated according to the method described by Wright [12].

### DNA sampling and analysis

Saliva is collected in the Oragene kit of DNA Genotek. DNA will be obtained from these kits, and subsequently we will perform whole genome SNP arrays by making use of existing platforms of SNP chips.

### Statistical Analysis

Using SNP markers, a genotype will be made for every individual from whom we obtained a saliva sample. Progeny will be used to generate the individual marker information. For the analysis, only independent markers will be used. Estimations of the inbreeding coefficient will be calculated by using the method as developed by Wang [13]. This method generates IBD estimates based on the observed 'identity-by-state' (IBS) sharing between the partners. For these calculations we plan to use free accessible software. If there is a difference between the couples in the calculated F-based on genealogical data - we will correct for this in our analysis.

The significance of the difference in estimated inbreeding coefficient based on genotype-data between our case couples and our control couples will be determined by using a paired t-test. If our data do not follow a (log)normal distribution, we will use the Wilcoxon matched pairs signed rank sum test.

## Discussion

This study's design contains a new molecular approach to the increased risk in consanguineous couples. In scientific research so far, homozygosity mapping in affected children of consanguineous couples has been used for finding causative genes. On the other hand, the above-mentioned study by Woods et al looked at the amount of autozygosity in children of consanguineous relationships. These authors did not investigate whether affected children had more homozygous DNA than healthy children of consanguineous couples who did not have affected children. As far as we know, no study has been done on the amount of DNA IBD in the parents, nor has this been used to assess their risk of having affected children. The results of this study will contribute to designing future research, such as the recruitment of a large - if possible, international - cohort of consanguineous couples before reproduction. This cohort will allow us to obtain different risk figure estimates for the different proportions of DNA IBD. Once such estimates are available, couples will be able to benefit from reproductive options when informed more precisely about their risk status.

A limitation in our study design is the definition of our control couples. We select couples who have at least three healthy children. The chance that a carrier couple will have three healthy children, is nevertheless still 42%. Ideally, we would only include couples with much more healthy children to diminish the risk of carrier couples among the parents. However, given the size of most present-day families, finding matched control families that have at least three healthy children will be challenging enough.

## Abbrevations

AR: Autosomal Recessive; F: Inbreeding Coefficient; IBD: Identical-By-Descent; IBS: Identical-By-State; SNP: Single Nucleotide Polymorphism;

## Competing interests

The authors declare that they have no competing interests.

## Authors' contributions

MT is responsible for the data collection and also drafted the manuscript. MT is supervised by LtK, MC and LH. LtK is the principal applicant for the grant. MC, PH and ZB are co-applicants. DK provided statistical advice. All authors have read and approved the current version of the paper.

## Pre-publication history

The pre-publication history for this paper can be accessed here:

http://www.biomedcentral.com/1471-2350/11/113/prepub
